# Antioxidant potential of curcumin-related compounds studied by chemiluminescence kinetics, chain-breaking efficiencies, scavenging activity (ORAC) and DFT calculations

**DOI:** 10.3762/bjoc.11.151

**Published:** 2015-08-11

**Authors:** Adriana K Slavova-Kazakova, Silvia E Angelova, Timur L Veprintsev, Petko Denev, Davide Fabbri, Maria Antonietta Dettori, Maria Kratchanova, Vladimir V Naumov, Aleksei V Trofimov, Rostislav F Vasil’ev, Giovanna Delogu, Vessela D Kancheva

**Affiliations:** 1 Institute of Organic Chemistry with Centre of Phytochemistry, Bulgarian Academy of Sciences, Acad. G. Bonchev str. bl. 9, Sofia 1113, Bulgaria; 2 Emanuel Institute of Biochemical Physics, Russian Academy of Sciences, Kosygina str. 4, Moscow 119334, Russian Federation; 3 CNR-Institute of Biomolecular Chemistry, Traversa La Crucca 3, I-07100 Sassari, Italy; 4 Moscow Institute of Physics and Technology, 9 Institutskiy per., Dolgoprudny, Moscow Region, 141700, Russian Federation

**Keywords:** antioxidant activity, chain-breaking efficiencies, chemiluminescence kinetics, DFT calculations, scavenging activity (ORAC)

## Abstract

This study compares the ability to scavenge different peroxyl radicals and to act as chain-breaking antioxidants of monomers related to curcumin (**1**): dehydrozingerone (**2**), zingerone (**3**), (2*Z*,5*E*)-ethyl 2-hydroxy-6-(4-hydroxy-3-methoxyphenyl)-4-oxohexa-2,5-dienoate (**4**), ferulic acid (**5**) and their corresponding *C*_2_-symmetric dimers **6–9**. Four models were applied: model 1 – chemiluminescence (CL) of a hydrocarbon substrate used for determination of the rate constants (*k*_A_) of the reactions of the antioxidants with peroxyl radicals; model 2 – lipid autoxidation (lipidAO) used for assessing the chain-breaking antioxidant efficiency and reactivity; model 3 – oxygen radical absorbance capacity (ORAC), which yields the activity against peroxyl radicals generated by an azoinitiator; model 4 – density functional theory (DFT) calculations at UB3LYP/6-31+G(d,p) level, applied to explain the structure–activity relationship. Dimers showed 2–2.5-fold higher values of *k*_A_ than their monomers. Model 2 gives information about the effects of the side chains and revealed much higher antioxidant activity for monomers and dimers with α,β-unsaturated side chains. Curcumin and **6** in fact are dimers of the same monomer **2**. We conclude that the type of linkage between the two “halves” by which the molecule is made up does not exert influence on the antioxidant efficiency and reactivity of these two dimers. The dimers and the monomers demonstrated higher activity than Trolox (**10**) in aqueous medium (model 3). A comparison of the studied compounds with DL-α-tocopherol (**11**), Trolox and curcumin is made. All dimers are characterized through lower bond dissociation enthalpies (BDEs) than their monomers (model 4), which qualitatively supports the experimental results.

## Introduction

Bioantioxidants have played an important role throughout the last decade in the protection of human health (as food additives) and in treatment of various human diseases (in a monotherapy at high concentrations or in a combination therapy with other drugs at low concentrations) [1–2]. For that reason it is of prime importance to design new bioantioxidants as synthetic analogues of natural ones as potential pharmaceutical and food ingredients. The present study compares the capacity of curcumin-related compounds to scavenge different free radicals and to act as chain-breaking antioxidants.

**Figure 1:**
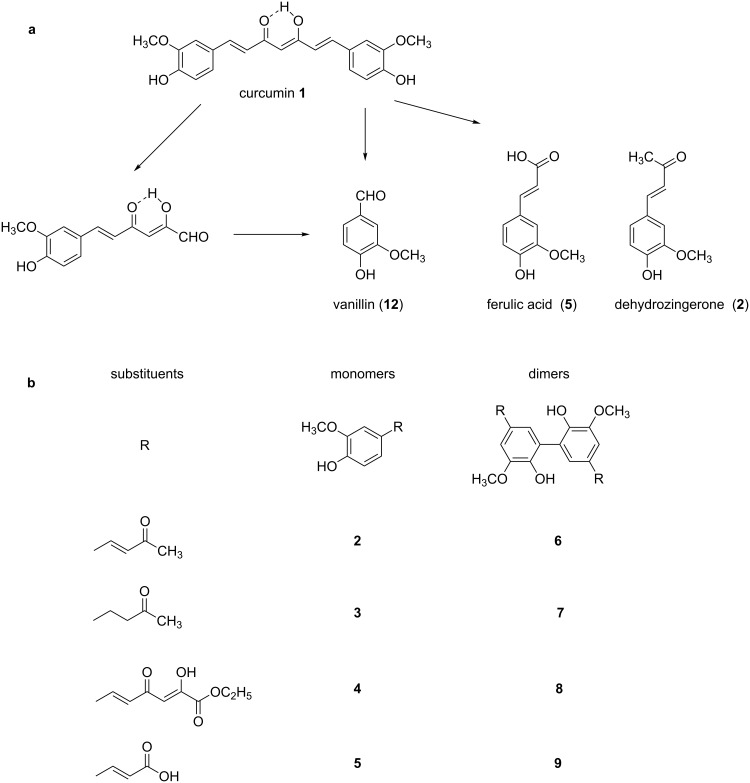
a) Degradation products of curcumin according to Wang et al. [10]; b) structures of the studied monomers/dimers.

Curcumin is one of the best natural antioxidants with a wide spectrum of biological activities [3–6]. It is known that curcumin can protect biomembranes against peroxidative damage mainly as scavenger of free radicals. Curcumin is a unique antioxidant, which contains a variety of functional groups [7]. It is generally assumed that the phenolic moieties are responsible for radical scavenging properties of the pertinent antioxidant reactants. The clinical use of curcumin is limited because of its low bioavailability, due to the hydrophobic nature of the molecule [8]. Schneider et al. [9] discussed the degradation and metabolism of curcumin, through products and their mechanism of formation. Its potential use in pharmaceutical applications and food industry is closely related to the stability of the compound. Curcumin is not stable under physiological conditions and its degradation products are depicted in Figure 1a [10].

With the aim of improving the chemical stability and biological activity of curcumin, hydroxylated biphenyls, which resemble the curcumin structure, were synthesized (Figure 1b). The most important features of their structure are: conformational flexibility, simplicity, reduced toxicity due to the presence of an *ortho*-methoxyphenol moiety, a sufficient number of favorable contacts with the protein compared to other aromatic compounds and, generally, stability under physiological conditions.

It was reported [11] that the dehydrozingerone dimer **6** partially inhibits the aggregation process of alpha synuclein (AS), which is involved as misfolded protein in Parkinson’s disease, while the zingerone dimer **7** interacts with high affinity with AS. Stability test showed that dimers **6** and **7** are stable at room temperature in phosphate-buffered solution or in the presence of AS or bovine serum albumin (20 mM, pH 6.8) between 98 and 100% over 36 min [12]. Interestingly, dimers **6** and **7** are nontoxic to PC12 cells at a concentration of 40 μM and, contrary to curcumin, dimer **7** protects PC12 cells against MnCl_2_ damage [11].

When the phenol OH groups of dimer **8** were protected with methyl or phenyl groups, antiproliferative activity against malignant melanoma cells was detected with IC_50_ values in the range of 8–15 μM, a concentration comparable to that of curcumin [13]. It is generally acknowledged that aryl β-diketoester moiety enolizes in the α-position to form the resultant stable *Z*-enol tautomer. The presence of a carbonyl function in conjugation with the enolic double bond allows the enol to be the predominant form. According to that, we decided to prepare biphenyl **8** and its corresponding monomer **4,** both featuring a stable *syn* keto–enol tautomeric form in the aliphatic side chains.

Large amounts of ferulic acid and the ferulic acid dimer **9** are present in saponified extracts of maize bran and grasses [14]. Ferulic acid and the corresponding ethyl ester protect primary neuronal cell cultures against oxidative damage [14]. The increased lipophilicity of ferulic acid ethyl ester improves the ability to cross cell membranes easier than the corresponding acid. The biphenylic structure provides lipophilicity to dimer **9** allowing for a strong effect against pests and plant diseases [15].

The different conformations of the biphenyl structure generated by selective functionalization of the aromatic rings led us to consider this moiety as a basic and valued framework for the synthesis of pharmacologically and agrochemically active new compounds.

The present study demonstrates experimental and theoretical models for assessing radical scavenging and antioxidant activity of curcumin-related hydroxylated biphenyls (dimers) and their corresponding monomers. Four models were applied: model 1 – chemiluminescence of a hydrocarbon substrate used for determination of the rate constants (*k*_A_) of the reactions of the antioxidants with peroxyl radicals (RO_2_**^•^**); model 2 – lipid autoxidation used for assessing the chain-breaking antioxidant efficiency and reactivity; model 3 – ORAC, which measures the scavenging activity against peroxyl radicals generated by an azoinitiator; and model 4 – a theoretical method used for predicting the activity of the studied compounds to scavenge free radicals by H-atom abstraction and to explain the structure–activity relationship of the studied compounds.

## Results and Discussion

**Scheme 1:**
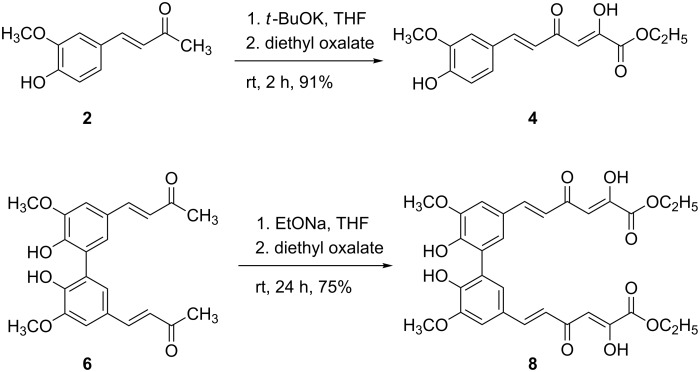
Preparation of hydroxylated biphenyl **8** and its monomer **4**.

Claisen condensation of biphenyl ketone **6** and monomer **2** with diethyl oxalate was carried out in the presence of a base to give β-diketo ethyl esters **8** and **4** in good yields (Scheme 1). All compounds prepared were solid and stable in air. *Trans-*configuration was exclusively obtained at the olefinic double bond and keto–enol form was observed in solution by NMR spectroscopy for esters **4** and **8**.

### (2*Z*,5*E*)-Ethyl 2-hydroxy-6-(4-hydroxy-3-methoxyphenyl)-4-oxohexa-2,5-dienoate (**4**)

Monomer **4** was obtained following the known procedure with slight modifications [16]: To a solution of potassium *tert*-butoxide (1.75 g, 15.6 mmol) in tetrahydrofuran (20 mL) was added dropwise a solution of dehydrozingerone (**2**, 1 g, 5.2 mmol) in THF (15 mL) at room temperature under N_2_ atmosphere. The reaction mixture was stirred at room temperature for 10 min. Diethyl oxalate (1.1 mL, 7.8 mmol) was added dropwise. The reaction mixture was stirred at room temperature for 2 h and then acidified with 10% HCl. The organic phase was extracted with CH_2_Cl_2_. The crude, dried over Na_2_SO_4_, gave a brown solid that was purified by flash chromatography using CH_2_Cl_2_ as eluent, to give **4** as an orange solid (1.38 g, 91%); mp 98–99 °C (lit [17]: 97–98 °C); ^1^H NMR δ 1.39 (t, *J* = 7.2 Hz, 3H), 3.95 (s, 3H), 4.36 (q, *J* = 7.2 Hz, 2H), 5.95 (bs, 1H), 6.51 (d, *J* = 15.6 Hz, 1H), 6.52 (s, =CH enol form, 1H), 6.94 (d, *J* = 8.4 Hz, Ar, 1H), 7.01 (d, *J* = 2.0 Hz, Ar, 1H), 7.14 (dd, *J* = 8.4, 2 Hz, Ar, 1H), 7.66 (d, *J* = 15.6 Hz, 1H); ^13^C NMR δ 14.07, 55.97, 62.45, 101.56, 109.66, 114.96, 120.69, 123.90, 126.99, 143.74, 146.89, 148.62, 162.26, 173.04, 185.94; Anal. calcd for C_15_H_16_O_6_: C, 61.64; H, 5.52; found: C, 61.72; H, 5.40.

### (2*Z*,2'*Z*,5*E*,5'*E*)-Diethyl 6,6'-(6,6'-dihydroxy-5,5'-dimethoxy-[1,1'-biphenyl]-3,3'-diyl)bis(2-hydroxy-4-oxohexa-2,5-dienoate) (**8**)

To a solution of sodium ethoxide (1.1 g, 15.7 mmol) in tetrahydrofuran (20 mL) was added dropwise a solution of **6** (1 g, 2.6 mmol) in THF (15 mL) at room temperature under an N_2_ atmosphere. The reaction mixture was stirred at room temperature for 10 min. Diethyl oxalate (0.96 mL, 6.5 mmol) was added dropwise. The reaction mixture was stirred at room temperature for 24 h and then acidified with 10% HCl. The organic phase was extracted with CH_2_Cl_2_. The crude, dried over Na_2_SO_4_, gave a brown solid that was purified by flash chromatography using a 9:1 mixture of petroleum/ethyl acetate as eluent, to give **8** as an orange solid (1.14 g, 75%); mp 192–193 °C; ^1^H NMR δ 1.38 (t, *J* = 7.2 Hz, 6H), 4.01 (s, 6H), 4.46 (q, *J* = 7.2 Hz, 4H), 5.92 (bs, 2H), 6.53 (d, *J* = 15.6 Hz, 2H), 6.55 (s, =CH enol form, 2H), 7.11 (d, *J* = 2.0 Hz, Ar, 2H), 7.24 (d, *J* = 2.0 Hz, Ar, 2H), 7.35 (d, *J* = 15.6 Hz, 2H); ^13^C NMR δ 14.04, 55.96, 62.44, 100.58, 109.76, 115.11, 120.59, 123.88, 126.94, 143.74, 146.99, 148.80, 162.22, 172.98, 185.95; Anal. calcd for C_30_H_30_O_12_: C, 61.85; H, 5.19; found: C, 61.99; H, 5.28.

### Model 1: Chemiluminescence with model hydrocarbon ethylbenzene

**Figure 2:**
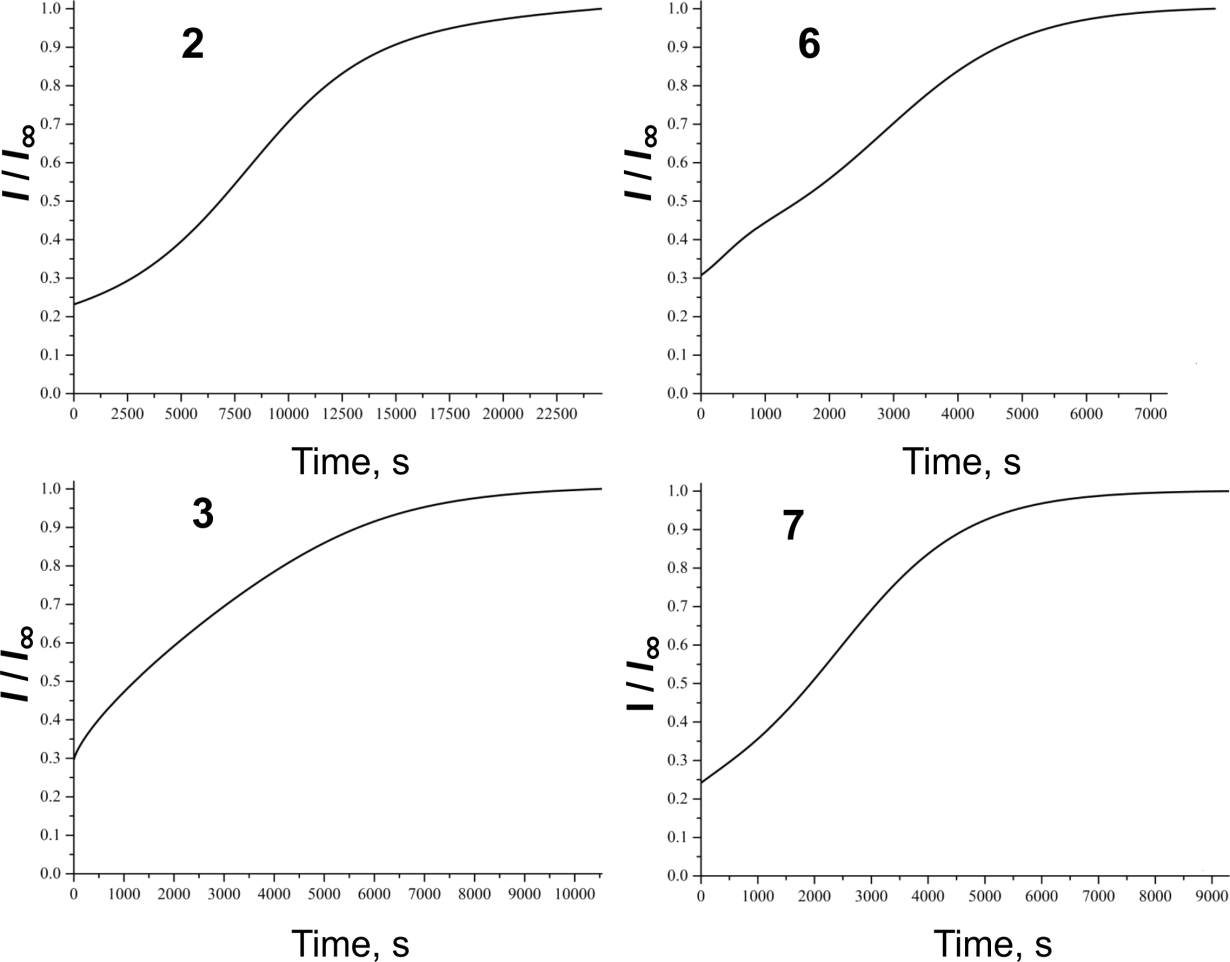
Time profiles of the relative chemiluminescence intensity (*I*/*I*_∞_) measured during the oxidation of ethylbenzene in aerated chlorobenzene solution (26 vol %) initiated by 2,2′-azobisisobutyronitrile (AIBN) at the rate of *R*_IN_ = 5.1 × 10^−9^ M·s^−1^ and 50 °C upon introduction of 1.2 × 10^−5^ M of the studied monomers/dimers.

**Figure 3:**
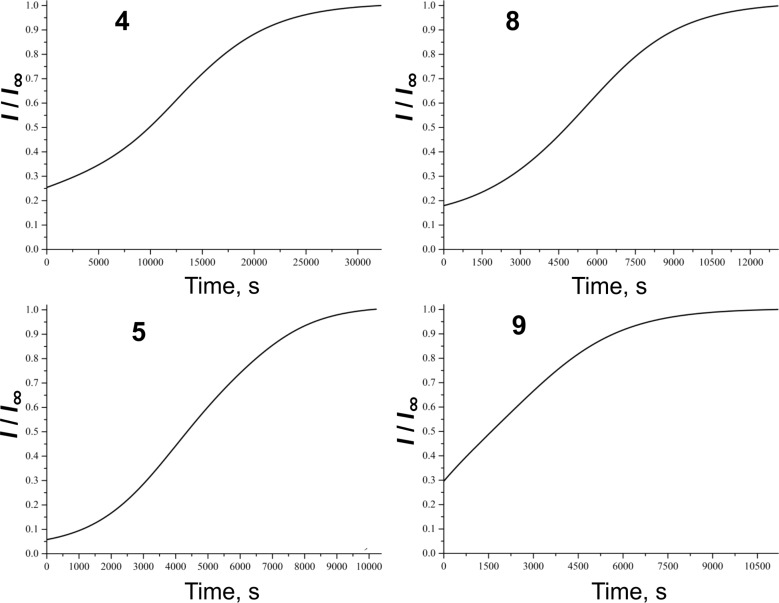
Time profiles of the relative chemiluminescence intensity (*I*/*I*_∞_) measured during the oxidation of ethylbenzene in aerated chlorobenzene solution (26 vol %) initiated by 2,2′-azobisisobutyronitrile (AIBN) at the rate of *R*_IN_ = 5.1 × 10^−9^ M·s^−1^ and 50 °C upon introduction of 1.2 × 10^−5^ M of the studied monomers/dimers.

The chemiluminescence time profiles upon introduction of antioxidants are displayed in Figure 2 and Figure 3. Processing these experimental data for all the antioxidants studied in the current work and according to Equation 1 (see Supporting Information File 1) the *k*_A_ values were calculated and listed in Table 1.

**Table 1:** Experimental obtained data by different models about the antioxidant characteristics of the studied monomers and dimers.

experimental model	model 1 CL^a^	model 2 lipidAO^b^		model 3 ORAC^c^

kinetic parameters	*k*_A_^d^ [M^−1^s^−1^]	PF^e^ [—]	ID^f^ [—]	RTE^g^ [—]

**2**	(1.7 ± 0.1) × 10^4^	3.5^h,i^	6.3^h,i^	3.12 ± 0.34
**6**	(4.2 ± 0.3) × 10^4^	13.5^h,i^	29.3^h,i^	3.04 ± 0.10
**3**	(2.6 ± 0.2) × 10^4^	3.5^h^	5.5^h^	3.85 ± 0.14
**7**	(5.1 ± 0.3) × 10^4^	5.8^h^	8.8^h^	2.97 ± 0.12
**4**	(2.6 ± 0.2) × 10^4^	5.8	9.8	5.60 ± 0.20
**8**	(3.4 ± 0.2) × 10^4^	12.8	11.0	5.22 ± 0.13
**5**	(1.6 ± 0.1) × 10^4^	3.2^h^	4.4^h^	2.56 ± 0.10
**9**	(4.1 ± 0.3) × 10^4^	3.3^h^	4.2^h^	1.98 ± 0.06
**11**	(1.0 ± 0.1) × 10^6^	21.2^i^	29.3^i^	—

^a^Chlorobenzene as medium, ethylbenzene as oxidizing substrate, AIBN (2,2′-azobisisobutyronitrile) as initiator at 50 °C; ^b^triacylglycerols of sunflower oil as oxidizing substrate, without an initiator, at 80 °C; ^c^water as medium, fluorescein (**13**) as substrate, AAPH *(*2,2'-azobisisobutyramidinium chloride) as initiator at 37 °C; ^d^rate constant *k*_A_ value acquired from the slope of the CL time profile at the inflection point according to (d*I*_rel_/d*t*)_max_ = 0.237(*k*_A_/(2*k*_t_)^0.5^)*R*_IN_; ^e^antioxidant efficiency, determined as protection factor (PF = IP_A_/IP_C_), where IP_A_ and IP_C_ are the induction periods in the presence and in the absence of an antioxidant; ^f^antioxidant reactivity as inhibition degree (ID = *R*_C_/*R*_A_), where *R*_C_ and *R*_A_ are the initial rates of lipid autoxidation in the absence and in the presence of the antioxidant; ^g^relative Trolox equivalent (RTE) was calculated according to the following equation (AUC: area under curve) [(AUC_Sample_ − AUC_Blank_)/(AUC_Trolox_ − AUC_Blank_)] × (molarity of Trolox/molarity of sample); ^h^from [19]; ^i^from [20].

It is seen from Table 1, that inhibition rate constants *k*_A_ for all the dimers and monomers are of the same order of magnitude and manifest a moderate antioxidant capacity, comparable with those of butylated hydroxytoluene (BHT). In this model DL-α-tocopherol (**11**) exhibits two orders of magnitude higher antioxidant potential with respect to the studied monomers/dimers, *k*_A_**^11^** = (1.0 ± 0.1) × 10^6^ M^−1^s^−1^. The values of *k*_A_ for **6**, **7** and **9** are 2–2.5-fold higher than those obtained for their corresponding monomers. This is indicative of the fact that both equal phenolic fragments in dimers act separately and independently of each other. Barclay et al. [18] studied the antioxidant mechanism of curcumin (**1**) and dehydrozingerone (**2**) (a half-curcumin molecule) in controlled regime of styrene oxidation in chlorobenzene. The authors observed that *k*_A_ value of **1** is twice as high as that of **2**. This result is in excellent agreement with our data obtained for the couple monomer **2**/dimer **6**. It might be concluded that it does not matter how the two half-curcumin moieties are combined: as curcumin **1**, or as *C*_2_-symmetric dimer **6**. Dimer **8** showed a 1.3-fold higher *k*_A_ value in comparison with monomer **4** probably due to the steric effects of the side chain that influences the dihedral angle of biphenyl structure.

### Model 2: Lipid autoxidation

**Figure 4:**
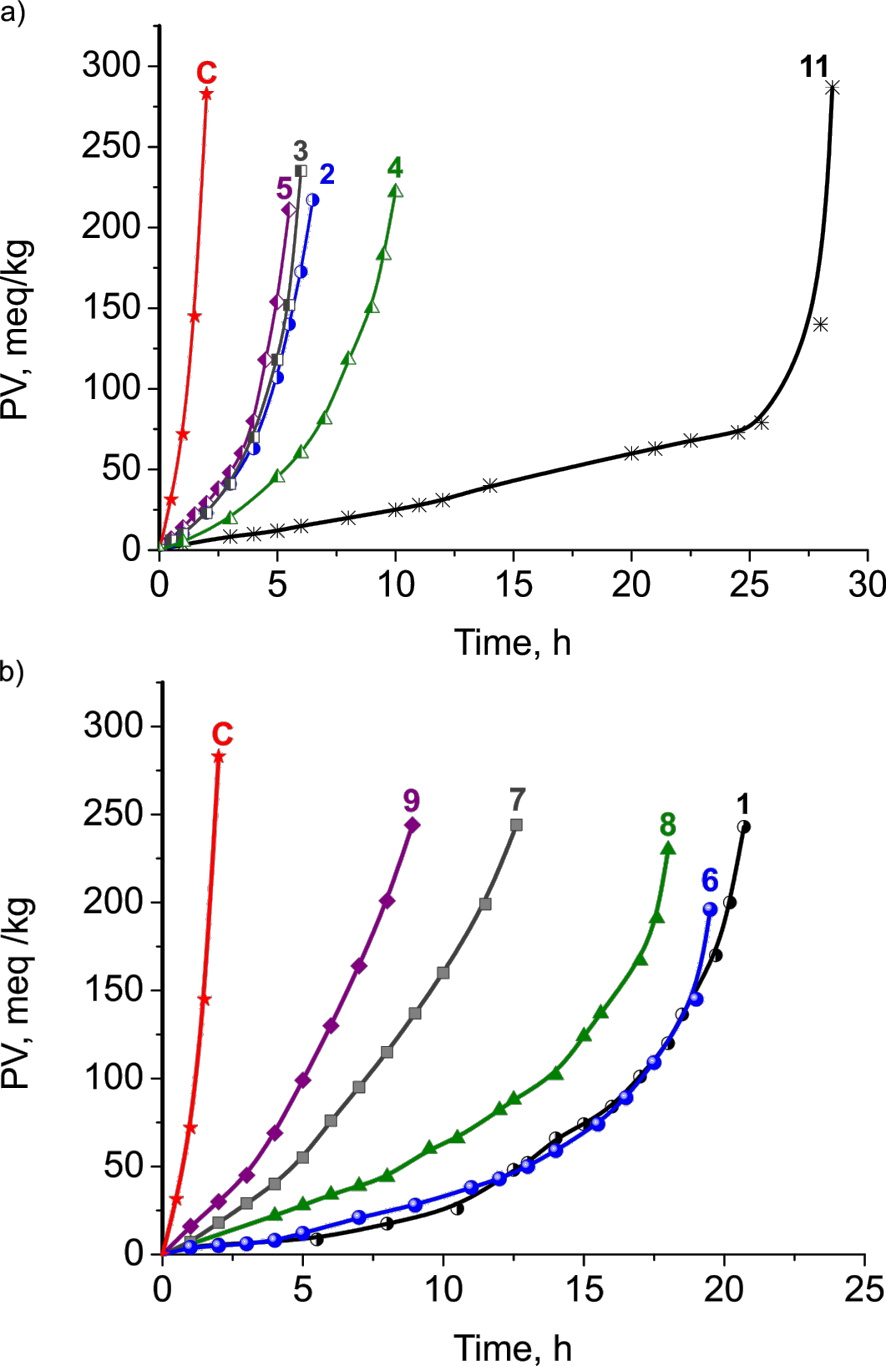
Kinetic curves of TGSO autoxidation at 80 °C in the absence (control, C) and in the presence of 1 mM of the studied compounds: a) monomers **2–5** and DL-α-tocopherol (**11**); and b) dimers **6–9** and curcumin (**1**).

Kinetic curves of the lipid autoxidation at concentrations of 1 mM of the studied compounds and of DL-α-tocopherol (**11**) are presented in Figure 4 and the main kinetic parameters, characterizing the autoxidation of triacylglycerols of sunflower oil (TGSO) are given in Table 1. Different orders of antioxidant efficiency (as protection factor, PF**,** see Supporting Information File 1 for details) and antioxidant reactivity (as inhibition degree, ID, see Supporting Information File 1 for details) were found:


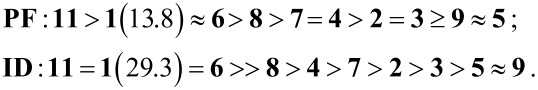


It is evident that in this model DL-α-tocopherol (**11**) is also the most efficient antioxidant, and ensures the highest oxidative stability of the lipid substrate. The observed lower antioxidant activity of monomers and dimers with respect to **11** is expected as a result of the higher BDE of phenolic –OH groups of these reactants. Another reason is that their phenoxyl radicals cannot be regenerated (such as those of **11**) through a homo-disproportionation reaction [21].

#### Comparison of dimers and monomers

It has been found that curcumin **1**, and the dimers **6** and **8** exhibited considerably stronger antioxidant efficiencies (PF) and inhibition degrees (ID) than the corresponding monomers **2** and **4**. Dimer **7** showed a slightly higher antioxidant efficiency and reactivity compared to its monomers **3** but a lower efficiency and reactivity relative to the dimers **6** and **8**.

The couples dimer **6**/monomer **2** and curcumin **1**/monomer **2** exhibited the biggest difference in the antioxidant efficiency (PF_d_/PF_m_ = 4) and antioxidant reactivity (ID_d_/ID_m_ = 5). Recently, it has been published [20] that, theoretically, one molecule of dimer **6** is able to trap maximum nine lipid peroxide radicals, LO_2_, i.e., the stoichiometry of **6** is *n* = 9 (4.5-fold higher than that of monomer **2**). This result explains the higher antioxidant efficiency obtained for dimer **6**, in comparison with the corresponding monomer **2**, and confirms the experimentally obtained four-fold higher PF (as antioxidant efficiency) of the dimer, which is due to a possible formation of various intermediates that are reactive towards LO_2_ radicals. Monomer **2** is a typical monophenolic antioxidant with stoichiometry *n* = 2 [19,22–24]. The couple dimer **6**/monomer **2** showed the greatest antioxidant activity as a result of the α,β-unsaturated ketone side chain in *para*-position of the phenolic –OH group, which is responsible for the resonance stabilization of the phenoxyl radicals formed and for the lowest level of side reactions. In case of curcumin, considered also as a dehydrozingerone dimer, the relative antioxidant kinetic parameters observed are almost the same as for the couple dimer **6**/monomer **2**.

A lower antioxidant potential of the couple dimer **8**/monomer **4** (PF_d_/PF_m_ = 2.2) and (ID_d_/ID_m_ = 1.1) was observed. This couple has a longer unsaturated side chain than dimer **6**/monomer **2**. Nevertheless, in both cases the resonance stabilization of the phenoxyl radical seems to be limited to the first oxygen atom of the keto–enol moiety. However, the results obtained proved that the presence of a longer side chain in the molecule of the studied compounds does not ensure a higher oxidation stability of the lipid substrate. Taking into consideration the way by which the two phenolic halves are linked together in the biphenyl molecule, i.e., in *ortho*-position to the phenol –OH group, a steric factor of the longer side chain exerts a different influence on monomer **4** and its corresponding dimer **8**.

Small differences in the relative kinetic parameters for the couple dimer **7**/monomer **3** (PF_d_/PF_m_ = 1.7 and ID_d_/ID_m_ = 1.6) observed can be rationalized in terms of the absence of an α,β-unsaturated ketone chain in *para*-position of benzene rings and thus lacking the resonance stabilization of the formed phenoxyl radicals.

There is no difference in the antioxidant efficiency (PF_d_/PF_m_ = 1.0) and reactivity (ID_d_/ID_m_ = 1.0) for the couple dimer **9**/monomer **5**. The unsaturated side chain is favorable for the resonance stabilization of phenoxyl radical. However, the –COOH group at the end of the side chain is able to accelerate the lipid oxidation through hydroperoxide decomposition into free radicals [24–27]. Dimer **9**, having two –COOH groups, displays a two-fold higher growing of the hydroperoxide decomposition rate. As a result, no higher antioxidant potential for dimer **9** compared to that of monomer **5** was observed.

Couples dimer **6**/monomer **2** and dimer **9**/monomer **5** are structurally similar and differ merely by the end of the side chain. The reported results showed that replacing –COOH with –COCH_3_ leads to an increase of the antioxidant potential of the dimer/monomer couple [24–27].

#### Comparison of dimer/dimer reactants

Dimer **6** and curcumin **1** showed the highest antioxidant reactivity, comparable with that of **11**, while dimers **7**, **8** and **9** are less efficient and reactive. A comparison of **6** with the other dimers showed: (i) a 2.0-fold higher PF and 3.3-fold higher ID compared to **7**; (ii) similar PFs of **6** and **8** and 2.7-fold higher ID with respect to **8**; (iii) a 4.0-fold higher PF and 7.0-fold higher ID of **6** in comparison with **9**.

Dimer **6** and curcumin **1** are dimers of the same monomer, namely dehydrozingerone (**2**). From the results obtained in model 2, i.e., under autoxidation conditions, we can conclude that the type of linkage between the two “halves” by which the molecule is made up does not exert any influence on the antioxidant efficiency and reactivity of the two dimers. The presence of the keto–enol moiety is not of significance for the hydrogen-atom-transfer (HAT) reactions and the classical chain-breaking antioxidant activity. The presence of two longer unsaturated keto–enol side chains in **8** leads to decrease in the activity, possibly, due to steric hindrance. However, the absence of a double bond in the side chains in **7** and the presence of –COOH groups at the end of the α,β-unsaturated chains in **9** causes a significant decrease in the activity of the compounds.

#### Comparison of monomers

The highest antioxidant efficiency was obtained for monomer **4**. It showed an almost 2-fold higher antioxidant efficiency and inhibition degree (1.7-fold higher PF and 1.6-fold higher ID than monomer **2**, 1.8-fold higher PF and ID than compared to monomer **3**, 1.8-fold higher PF and 2.3-fold higher ID than monomer **5**). These results may be rationalized in terms of the steric effect of the longer side chain in monomer **4**, which leads to a lower level of side reactions of the pertinent phenoxyl radicals. Monomers **2**, **3** and **5** demonstrated similar antioxidant efficiencies (PF). However, they differ in inhibition degree (ID), i.e., in their possibility to shorten the oxidation chain length. This result can be explained with the higher reactivity of phenoxyl radicals of monomers **3** and **5**, due to the absence of an α,β-unsaturated ketone chain in *para*-position of the phenolic –OH group (in case of **3**) and due to the presence of the –COOH group at the end of the side chain (in case of **5**).

### Model 3: Oxygen radical absorbance capacity assay with fluorescein

**Figure 5:**
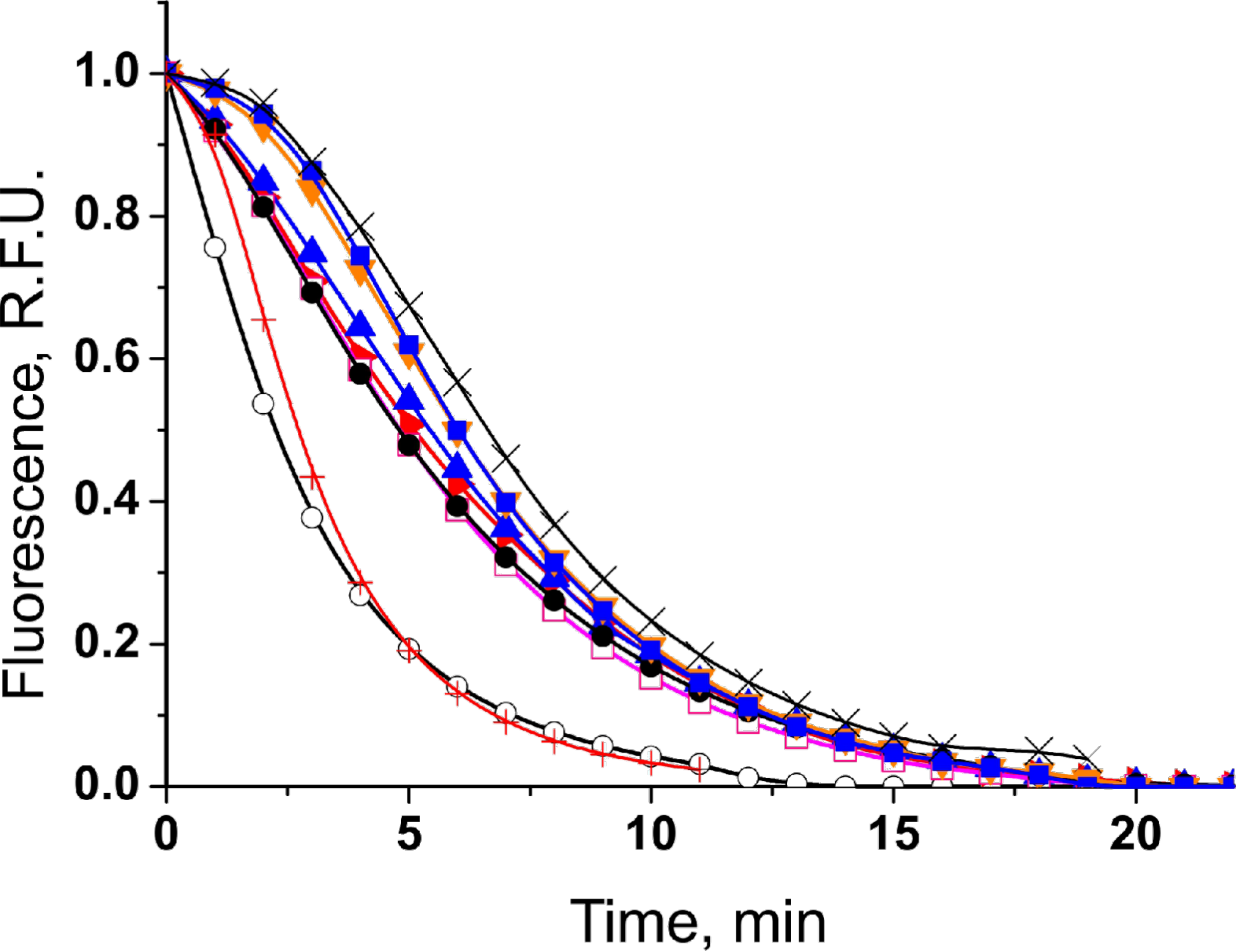
Fluorescence decay curves of fluorescein (**13**) in the absence (blank sample: white circles) and in the presence of the reference antioxidant Trolox (**10**) at 0.63 (red plus signs) and 2.50 μM (black cross signs), and of studied monomers **2** (red triangles), **3** (black circles), **4** (blue squares) and their dimers **6** (blue triangles)**, 7** (open pink squares) and **8** (orange triangles) at concentration 0.50 μM.

Figure 5 displays the kinetics of fluorescence decay for all studied monomers and dimers. The studied dimers exhibit activities that are similar or even lower compared to the corresponding monomers (Figure 5). Moreover, all compounds are less active than the reference antioxidant Trolox (**10**) when the latter is at 5-fold higher concentration (2.50 μM) but reveal the higher activity at a similar concentration of **10** (0.63 μM). It should be noted that the kinetic solvent effect of water is of great importance in this case. Hydrogen bonding can have a profound influence on the activity of phenols as antioxidants. It is generally recognized [28–30] that solvent effects and especially the ionization effect of the medium may alter the mechanism of the antioxidant action. However, the ORAC assay is a method for the detection of radical-scavenging activity based on the HAT mechanism [31–32]. From the results obtained (see Table 1) in aqueous medium there are no considerable differences in the antioxidant activities between the monomers and dimers in terms of the HAT reaction mechanism. This could be explained by the solvent effect of water, blocking OH groups by H bonds and thus decreasing their antioxidant capacity.

Another reason for the similar results obtained for the studied monomers/dimers is the fact that the process is monitored by the fluorescence decay of fluorescein (**13**). In this model, there are competitive reactions of peroxide radicals between **13** and the studied compounds. The first attack in **13** is its phenolic group (see Scheme S4, Supporting Information File 1). As a monophenol without another substituents in the benzene ring, we expected **13** to be slightly less active than the studied *ortho*-methoxy substituted monomers and dimers. According to Huang et al. [32] if the condition 100 *k*_FL_[fluorescein] < *k*_Am_[monomer] (k_Ad_[dimer]) is met, the rate of inhibited oxidation should be two orders of magnitude lower than the rate of uninhibited oxidation one and a lag phase would be observed. We did not observe any lag phase in the presence of studied monomers and dimers at a 10-fold higher concentration in comparison to that of **13**. This means that there should not be considerable differences between *k*_FL_, *k*_Am_, and *k*_Ad_. The quantum chemical calculations (see section “Model 4: DFT calculations”) for BDE of **13** and studied compounds in water medium confirm our hypothesis.

It is noteworthy that the literature data for individual phenolic compounds acquired with ORAC as a model system are rather poor in comparison with the data reported for different plant extracts.

### Model 4: DFT calculations

In order to explain the structure–activity relationships of the hydroxylated biphenyls and their corresponding monomers, we have optimized the geometries of the compounds and possible phenoxyl radical species of the parent compounds. The UB3LYP/6-31+G(d,p) optimized structures of compounds and their radicals in the gas phase are given in Table 2.

**Table 2:** Optimized structures, sums of electronic and thermal enthalpies (*H*_298_, a.u.) of the studied compounds, dihedral angles (φ, °) of the dimers and bond dissociation enthalpies (BDE, kcal·mol^−1^). The values calculated for the structures optimized in water are given in parentheses.

compound	molecule	radical	biradical	BDE, kcal·mol^−1^

**2**	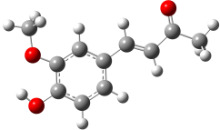 *H*_298_ = −651.868596 (−651.878465)	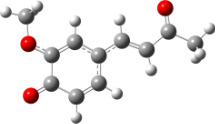 *H*_298_ = −651.241585 (−651.255413)	—	79.53 (77.04)
**6**	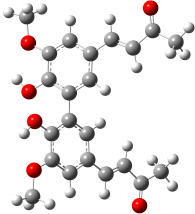 *H*_298_ = −1302.554462 (−1302.573379) φ = 64.576 (68.112)	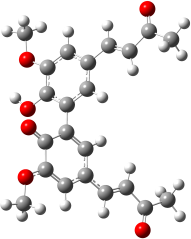 *H*_298_ = −1301.928383 (−1301.950845) φ = 63.311 (64.932)	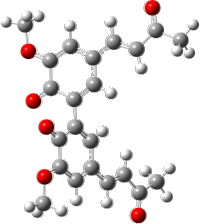 *H*_298_ = −1301.301389 (−1301.328123) φ = 68.849 (67.146)	(r) 78.94 (76.71) (br) 79.52 (76.83)
**3**	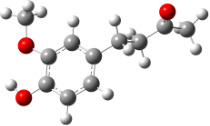 *H*_298_ = −653.066142 (−653.075843)	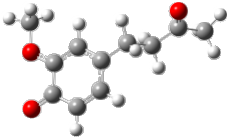 *H*_298_ = −652.438022 (−652.453157)	—	80.23 (76.81)
**7**	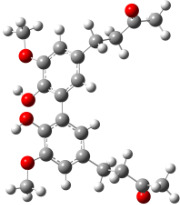 *H*_298_ = −1304.948860 (−1304.968218) φ = 62.231 (68.021)	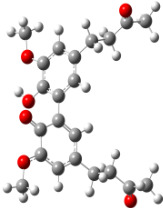 *H*_298_ = −1304.321729 (−1304.346187) φ = 59.741 (64.752)	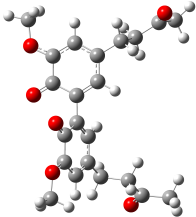 *H*_298_ = −1303.693274 (−1303.724652 φ = 69.640 (67.224)	(r) 79.60 (76.40) (br) 80.44 (76.09)
**4**	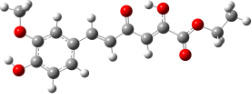 *H*_298_ = −1032.317386 (−1032.405066)	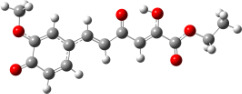 *H*_298_ = −1031.689944 (−1031.705790)	—	79.80 (77.56)
**8**	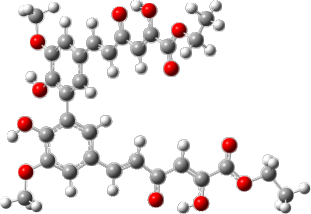 *H*_298_ = −2063.452015 (−2063.475877) φ = 63.073 (66.675)	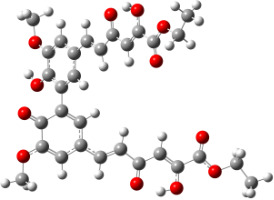 *H*_298_ = −2062.825553 (−2062.852556) φ = 61.652 (64.879)	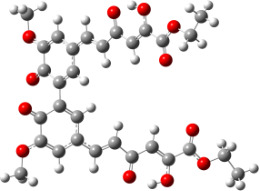 *H*_298_ = −2062.198378 (−2062.228955) φ = 66.802 (65.673)	(r) 79.18 (77.21) (br) 79.63 (77.38)
**5**	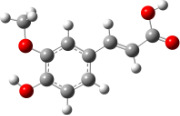 *H*_298_ = −687.821096 (−687.831734)	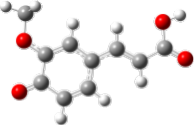 *H*_298_ = −687.193195 (−687.207615)	—	80.09 (77.71)
**9**	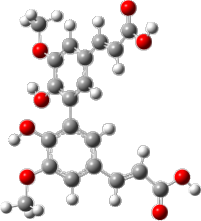 *H*_298_ = −1374.461690 (−1374.481055) φ = 64.582 (67.996)	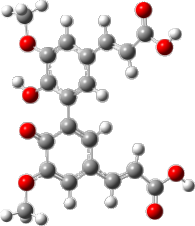 *H*_298_ = −1373.834836 (−1373.857451) φ = 62.755 (66.047)	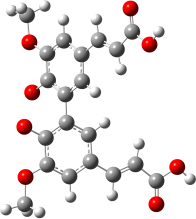 *H*_298_ = −1373.207082 (−1373.233750) φ = 67.994 (66.845)	(r) 79.43 (77.39) (br) 80.00 (77.45)
**1 (enol)**	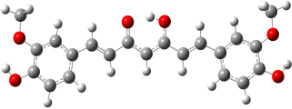 *H*_298_ = −1263.272328 (−1263.286825)	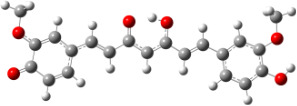 *H*_298_ = −1262.646509 (−1262.664892)	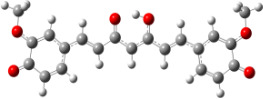 *H*_298_ = −1262.019583 (−1262.041985)	(r) 78.78 (br) 79.48
**11**	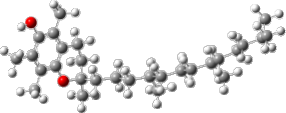 *H*_298_ = −1285.030320	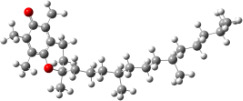 *H*_298_ = −1284.417253		70.78
**10**	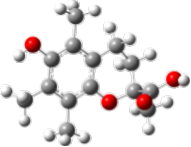 (*H*_298_ = −845.002438)	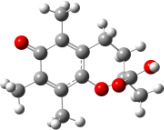 (*H*_298_ = −844.389866)		(70.46)
**13**	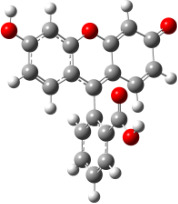 *H*_298_ = −1145.250604 (−1145.270685)	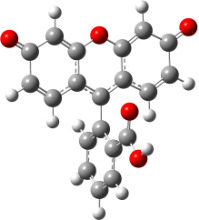 *H*_298_ = −1144.625264 (−1144.643325)		78.48 (79.74)

**Figure 6:**
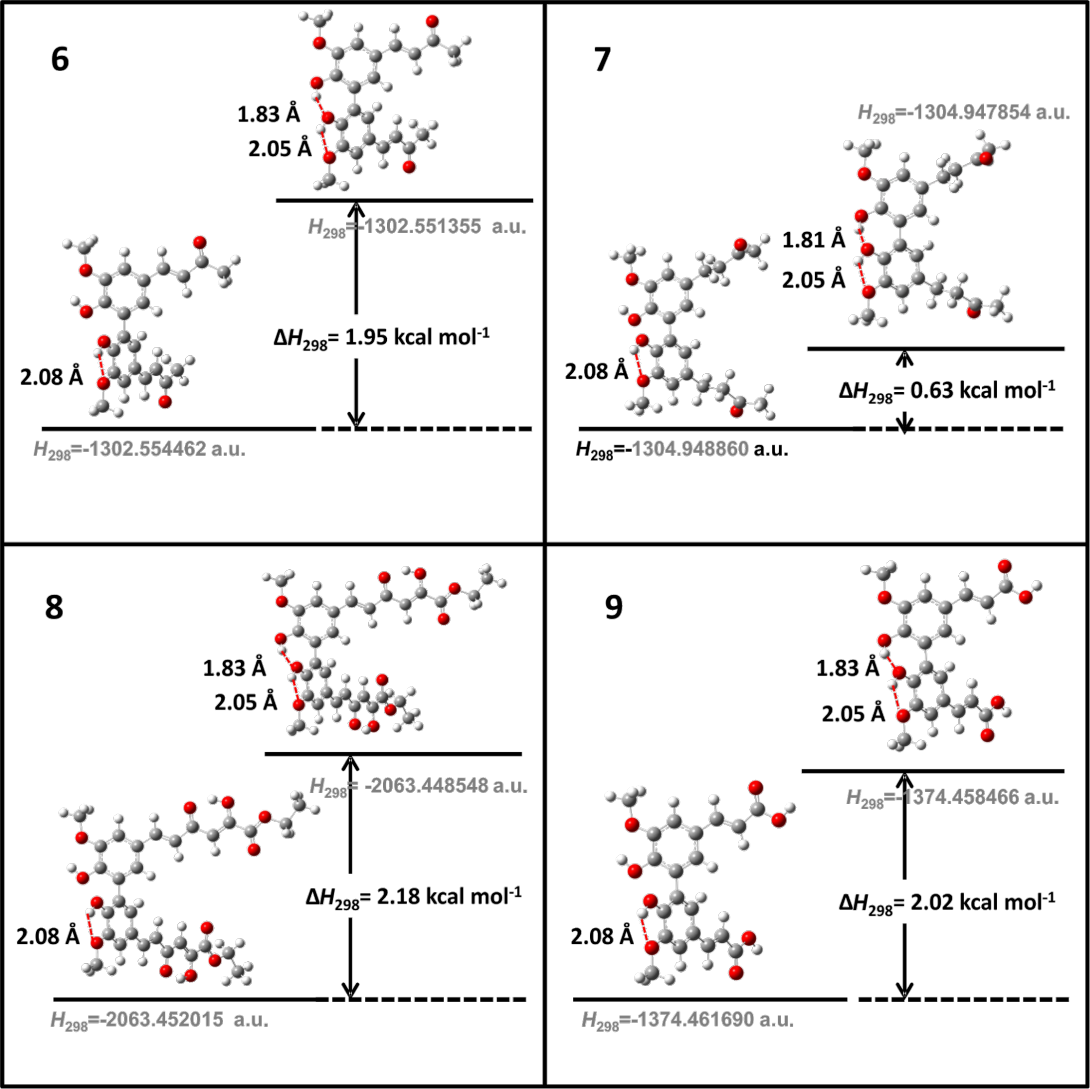
B3LYP/6-31+G(d,p)-optimized structures of the dimers and enthalpy differences between dimers with and without H bonds between –OH groups from both rings. The lengths of the H bonds are also given.

For all monomers only rotamers with intramolecular hydrogen bonds between the hydroxy and the methoxy groups (O atom) are considered. In case of monomer **4** the structures with hydrogen bonds between keto and enol groups are the objects of our investigation. Possible structures for the hydroxylated biphenyls are (i) with hydrogen bonds between hydroxy and methoxy groups and (ii) with hydrogen bonds between both –OH groups. These structures and their relative enthalpies are presented in Figure 6. The structures without H bond between both rings are favored in all cases. The enthalpy differences for dimers **6**, **8** and **9** are very close, in the range of 1.95–2.18 kcal·mol^−1^, while the enthalpy difference for dimer **7** (the only compound with saturated side chains) is only 0.63 kcal·mol^−1^ due to the higher intrinsic flexibility. The structures with the H bond between both rings are excluded from the further consideration because of the higher enthalpy (*H*_298_) and distortion of the 7-membered ring formed in these compounds.

Quantum chemical parameters characterizing the neutral molecules and their possible radicals/biradicals are collected in Table 2. For the monomers similar BDE values are found (the biggest difference is 0.7 kcal·mol^−1^, i.e., lower than 1 kcal·mol^−1^), i.e., **2–4** should manifest similar activity and close to that of ferulic acid **5**. Two BDE values are calculated for the biphenyls in accordance with the ability to form radical (r) and biradical (br) species. The BDE of biradicals is equal or very close to the BDE value of the corresponding monomers, whereas the BDE of radicals is smaller than the BDE of biradicals with differences in the range of 0.45–0.84 kcal·mol^−1^. These values could explain qualitatively the experimentally derived higher antioxidant activity and radical scavenging activity of dimers compared to monomers. Compounds with an α,β-unsaturated ketone chain are characterized by lower BDEs than those with saturated side chains. A carboxy group at the end of the α,β-unsaturated side chains (**5** and **9**) leads to slightly higher (about 0.56 kcal·mol^−1^) BDE values in comparison with monomer **2** and dimer **6**.

The changes in the dihedral angles between the benzene rings of the dimers **6–9** are similar: The formation of the radical (slightly) decreases the angle while the subsequent formation of the biradical increases the angle more noticeably. The values of the dihedral angles of **7** differ significantly between the radical and the biradical species, probably due to the higher conformational flexibility of the structure in virtue of the presence of two chains with saturated bonds.

**Figure 7:**
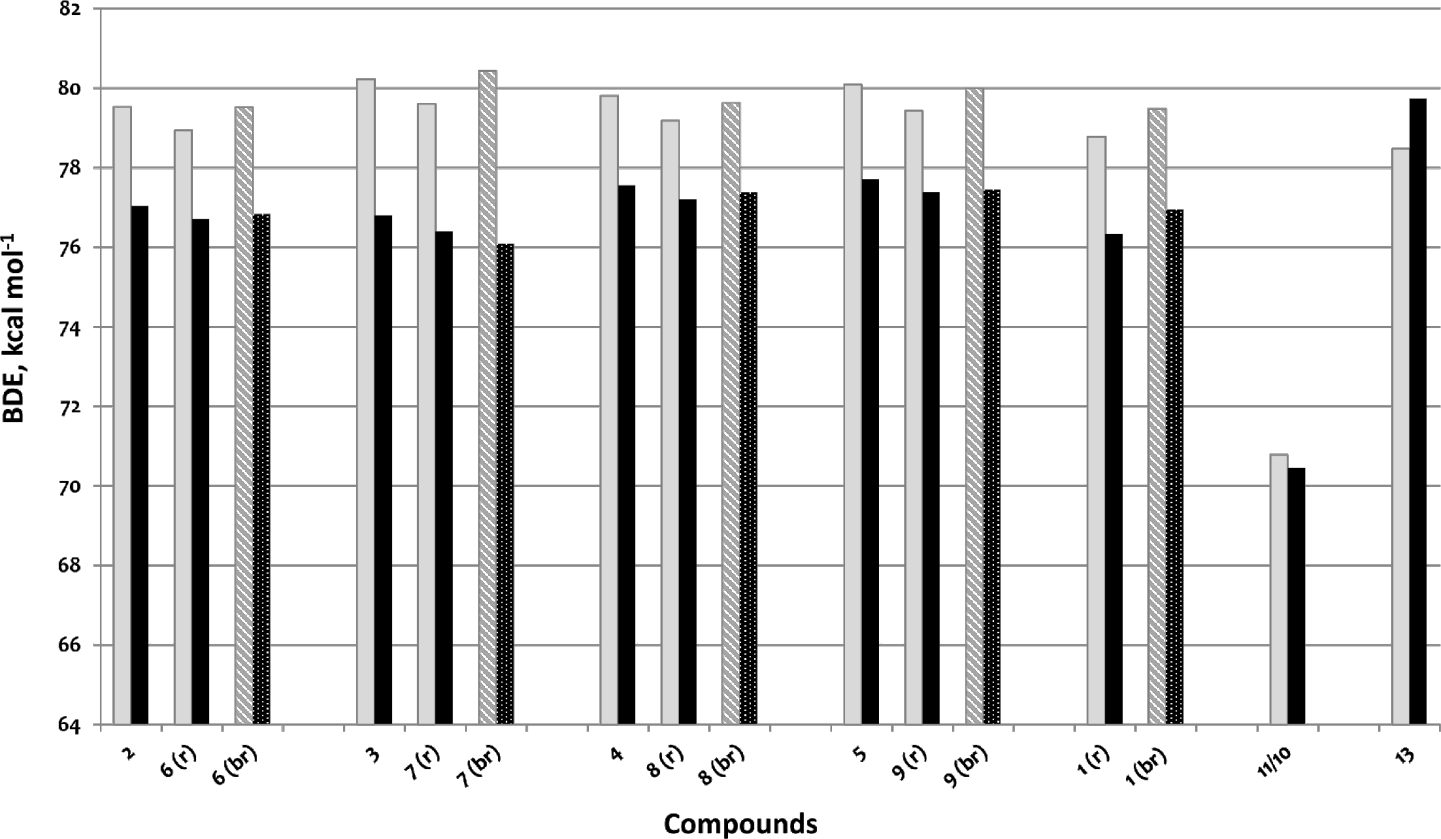
Bond dissociation enthalpies (BDEs). Solid fill refers to monomers and radicals in gas phase (grey) and in water (black); the pattern fill refers to biradicals in gas phase (diagonally striped pattern) and in water (dot pattern).

In order to take into account the effect of the solvent as dielectric medium all structures were optimized in water. The results are listed in Table 2 and presented graphically on Figure 7. In water medium all compounds are characterized by lower BDE values. The BDEs of monomer **4**/dimer **8** and monomer **5**/dimer **9** are very close in water. The changes are most pronounced for the couple **3**/**7,** and **3** became the monomer with lowest BDE value and **7** the dimer with the lowest BDE value. The biradical of dimer **7** possesses a BDE value lower than that of the radical formed from this compound. The tendency in the dihedral angles between the benzene rings in the dimers is also different in water. The initial molecules are more twisted, the formation of the radical decreases the angle noticeably and the subsequent formation of the biradical increases the angle again.

For comparison, in Table 2 and in Figure 6 are presented data for **1**, **10**, **11** and **13**. In gas phase **6** has a BDE(r) value close to that of curcumin (**1**) (78.94 and 78.78 kcal·mol^−1^, respectively), which is in agreement with the experimental data. In water the BDE(r) values of **7** and of **6** are almost equal (76.40 and 76.71 kcal·mol^−1^). In gas phase **13** is characterized with BDE value in the range of that of the monomers, while in water higher value was evaluated. The BDE values calculated for **11** in gas phase and for **10** in water are much lower than the values determined for the compounds under study. This explains the experimentally obtained results only in aprotic media.

### Comparative analysis of the results obtained by using different models and structure–activity relationship

The initiated oxidation of a model hydrocarbon substrate (model 1) has an advantage that in this system the rate of initiation (*R*_IN_) is well controlled (constant value) and the contribution of side reactions is minimal and can be neglected. For that reason, model 1 enables obtaining the absolute value of inhibition rate constant *k*_A_ (the key reaction of inhibited oxidation). It is noteworthy that the *k*_A_ values of dimers **6** and **9** are equal as a result of their similar structures. Dimer **7** exhibited the highest *k*_A_ value, and this result nicely correlates with scavenging towards the DPPH radical reported by Kancheva et al. [19]. The *k*_A_ value of dimer **8** is a bit lower than that of dimer **6**, probably due to steric factors. In general, there are no significant differences among all four dimers tested, and this is in a good agreement with the theoretical BDE values, which are rather close.

In contrast to the initiated oxidation, during lipid autoxidation (model 2) the rate of initiation increased with time as a result of accelerated hydroperoxide decomposition and of significant role of side reactions (see Scheme S3, Supporting Information File 1). Contrary to model 1, model 2 deals with the superimposed effects of all the oxidation steps. As a result the effects of small differences in side chains are essential for the main kinetic parameters of lipid autoxidation. Influence of side reactions is evident from the increase of the ID value in case of **9** in comparison to that of dimer **6** (ID_6_/ID_9_ = 7). In model 1, the *k*_A_ values of dimers **6** and **9** are similar because of the initiated oxidation and the lack of side reactions. In case of **7** a 3-fold decrease of the ID value was observed in comparison with **6**. The couples dimer **8**/monomer **4** and curcumin **1**/monomer **2** exhibited the greatest antioxidant activity as a result of the α,β-unsaturated ketone chain in *para*-position to the –OH group in the benzene rings, which is responsible for a possible resonance stabilization of the phenoxyl radicals formed and the lowest influence of side reactions. The results obtained proved that the longer side chain does not increase the antioxidant efficiency and/or reactivity of the couple monomer **4**/dimer **8**. The reported results showed that replacing of –COOH with –COCH_3_ leads to increase of the antioxidant potential of the dimer/monomer couple.

Model 3 compares the effects of monomers/dimers in the presence of an initiator (which is similar to model 1), but in aqueous medium. In this case similar antioxidant potentials for monomers and dimers and lower activity of Trolox (**10**) with respect to the studied monomers/dimers were encountered. The latter observation may be accounted for by a solvating effect of water derived from hydrogen bonding between the OH groups and water molecules. In contrast to models 1 and 2, where **11** is the best antioxidant, in model 3 the water soluble analogue of **11** (**10**) is less active than monomers/dimers at the same concentration or with similar activity at 5-fold higher concentration.

Model 4 has revealed that the substantial differences in the geometries of the studied series of monomers and dimers are not the case. Gas-phase calculated BDEs lie in relatively narrow range, i.e., differ slightly, including the values calculated for curcumin. The differences in the monomer/dimer couples are approximately identical, so that minimal influence of the side chain can be supposed. All dimers have lower BDEs than the corresponding monomers, which is in perfect qualitative correlation with models 1 and 2. The similar activity of the studied compounds acquired with the ORAC assay can be explained with the similar BDEs of **13** and of monomers and dimers.

## Conclusion

This study compares the ability of curcumin-related compounds to scavenge different peroxide radicals and to act as chain-breaking antioxidants. The experimental results (models 1–3) obtained are qualitatively supported by quantum chemical calculations (model 4).

Dimers showed higher activity than the corresponding monomers in the experiments in homogeneous solutions (models 1 and 2) and similar activity in the presence of water (model 3). The presence of an α,β-unsaturated chain seems to be a key factor in chain-breaking antioxidant activity of the studied compounds, likely due to a stronger resonance stabilization of the generated radical. It is obtained for the first time that dimers and monomers demonstrated the higher activity in comparison with Trolox in water medium (model 3). The zingerone dimer **7**, the strongest scavenger of free radicals, is not able to inhibit effectively the lipid autoxidation (model 2), because of the lack of an α,β-unsaturated ketone chain in *para*-position to phenolic –OH group. Thus, no effective stabilization of the phenoxyl radicals occurs. Although small differences in the BDE values between monomers and dimers have been found, the theoretical results are in reasonable agreement with the experimental data. Dimer **6** and curcumin **1** are dimers of the same monomer, namely dehydrozingerone (**2**). In conclusion, from the results obtained we can conclude that the type of linkage between the two “halves” by which the molecule is made up does not exert influence on the antioxidant efficiency and reactivity of the two dimers.

## Supporting Information

Experimental data (materials, methods, instruments and procedures), structural characterization of the synthesized compounds **2**, **6**, **7**, **9**, details for the four models applied to assess the antiradical and antioxidant activities of the compounds studied.

File 1 Additional experimental and theoretical data.
