# Supplementary material for: Antioxidant potential of curcumin-related compounds studied by chemiluminescence kinetics, chain-breaking efficiencies, scavenging activity (ORAC) and DFT calculations
Source: Beilstein J Org Chem. 2015 Aug 11;11:1398–411. doi: 10.3762/bjoc.11.151 (PMC4578382; doi:10.3762/bjoc.11.151)
Supplement: File 1 — Additional experimental and theoretical data. [file Beilstein_J_Org_Chem-11-1398-s001.pdf]

Supporting Information

for

Antioxidant potential of curcumin-related compounds studied by chemiluminescence kinetics, chain-breaking efficiencies, scavenging activity (ORAC) and DFT calculations

Adriana K. Slavova-Kazakova[1], Silvia E. Angelova[1], Timur L. Veprintsev[2], Petko Denev[1], Davide Fabbri[3], Maria Antonietta Dettori[3], Maria Kratchanova[1], Vladimir V. Naumov[2], Aleksei V. Trofimov[2,4], Rostislav F. Vasil'ev[2], Giovanna Delogu*[,§3], Vessela D. Kancheva*[,‡1]

Address: [1]Institute of Organic Chemistry with Centre of Phytochemistry, Bulgarian Academy of Sciences, Acad. G. Bonchev str. bl. 9, Sofia 1113, Bulgaria; [2]Emanuel Institute of Biochemical Physics, Russian Academy of Sciences, Kosygina str. 4, Moscow 119334, Russian Federation; [3]CNR-Institute of Biomolecular Chemistry, Traversa La Crucca 3, I-07100 Sassari, Italy and [4]Moscow Institute of Physics and Technology, 9 Institutskiy per., Dolgoprudny, Moscow Region, 141700, Russian Federation

Email: Vessela D. Kancheva[*] - vedeka@abv.bg; Giovanna Delogu[*] - giovanna.delogu@icb.cnr.it

Corresponding authors:

[§]Phone: +39079 2841220; Fax: + 39079 2841299

[‡]Phone: +359 29606187; Fax: + 359 28700225

Additional experimental and theoretical data

Contents

1. **Materials and methods.** S2

1.1. **Synthesis of C_2-symmetric hydroxylated biphenyls 6, 7, 9 and of monomer 2** S3

1.2. **Spectral characteristics of the studied compounds** S4

2. **Studies of antioxidant potential by four models** S4

2.1. **Model 1. Estimation of rate constant of antioxidant reaction with peroxyl radicals (k_A) using kinetic chemiluminescence (CL) method** S4

2.2. **Model 2. Estimation of the chain-breaking antioxidant activity of the studied compounds during lipid autoxidation** S6

2.3. **Model 3. Estimation of oxygen radical absorbance capacity (ORAC)** S8

2.4. **Model 4. DFT calculations** S9

References S10

1. Materials and methods

DL-α-Tocopherol (**11**) was obtained from (E. Merck, Germany), Trolox (**10**) (the water soluble analogue of **11**), and curcumin (**1**) were purchased from Sigma-Aldrich Chemie (Steinheim, Germany). Zingerone (**3**) was purchased from Chemos GmbH (Regenstauf, Germany).

Starting materials (vanilin (**12**), zingerone (**3**), ferulic acid (**5**)), reagents and solvents were obtained from commercial suppliers (Sigma-Aldrich Chemie GmbH and E. Merck, Germany) and were used without further purification. Acetone was freshly distilled from $CaCl_2$, tetrahydrofuran (THF) from sodium wire. Melting points were determined on a Büchi 530 apparatus (Assago, Italy) and are uncorrected. All ^{1}H and ^{13}C NMR spectra were recorded in $CDCl_3$ and DMSO-d_6 solutions with a Varian Mercury Plus (Palo Alto, CA) spectrometer at 400 MHz and 100.57 MHz, respectively. Chemical shifts (δ) are given in ppm; multiplicates are indicated by s (singlet), d (doublet) or dd (double of doublets). Elemental analyses were performed using an elemental analyzer Perkin-Elmer model 240 C (Waltham, Massachusetts). Flash chromatography was carried out with silica gel 60, 230–400 mesh (Aldrich, Milano, Italy) eluting with appropriate solution in the stated v:v proportions. Analytical TLC was performed with either 0.25 mm thick silica gel plates (Polygram® SilG/UV_{254}, Macherey-Nagel, VWR International, Italy) or 0.2 mm thick silica gel plates (60 5254 Merck, Italy). The purity of all new compounds was judged to be >98% by ^{1}H NMR and ^{13}C NMR spectral determination. Monomers

2 and **3** and dimers **6** and **7** were previously prepared by us following straightforward methods as depicted in Scheme S1 [s1]. Dimer **9** was prepared according to the known procedure [s2].

1.1. Synthesis of C_2-symmetric hydroxylated biphenyls 6, 7, 9 and of monomer 2

Biphenyl **7** was prepared in 65% yield by C–C coupling of zingerone (**3**) in the presence of methyl tributyl ammonium permanganate (MTBAP) in dichloromethane at room temperature for 1 h (Scheme S1). Unfortunately, the procedure turned out to be unsuccessful in preparation of dimer **6** that was obtained in 83% yield by Claisen–Schmidt condensation of vannilin dimer **14** in the presence of large excess of LiOH in acetone at room temperature. With a slight modification of the above synthetic procedure, dehydrozingerone (**2**) was achieved starting from vanillin (**12**) in acetone using NaOH as base. Dimer **9** was prepared by Perkin reaction of diacetate dimer of vanillin (**15**) in the presence of malonic acid and bases and further hydrolysis of the acetate groups. All compounds prepared were solid, air-stable and they were fully characterized. In the synthesis of unsaturated compounds **2**, **6**, and **9** *trans*-configuration was exclusively obtained at the olefinic double bond and detected by NMR spectroscopy.

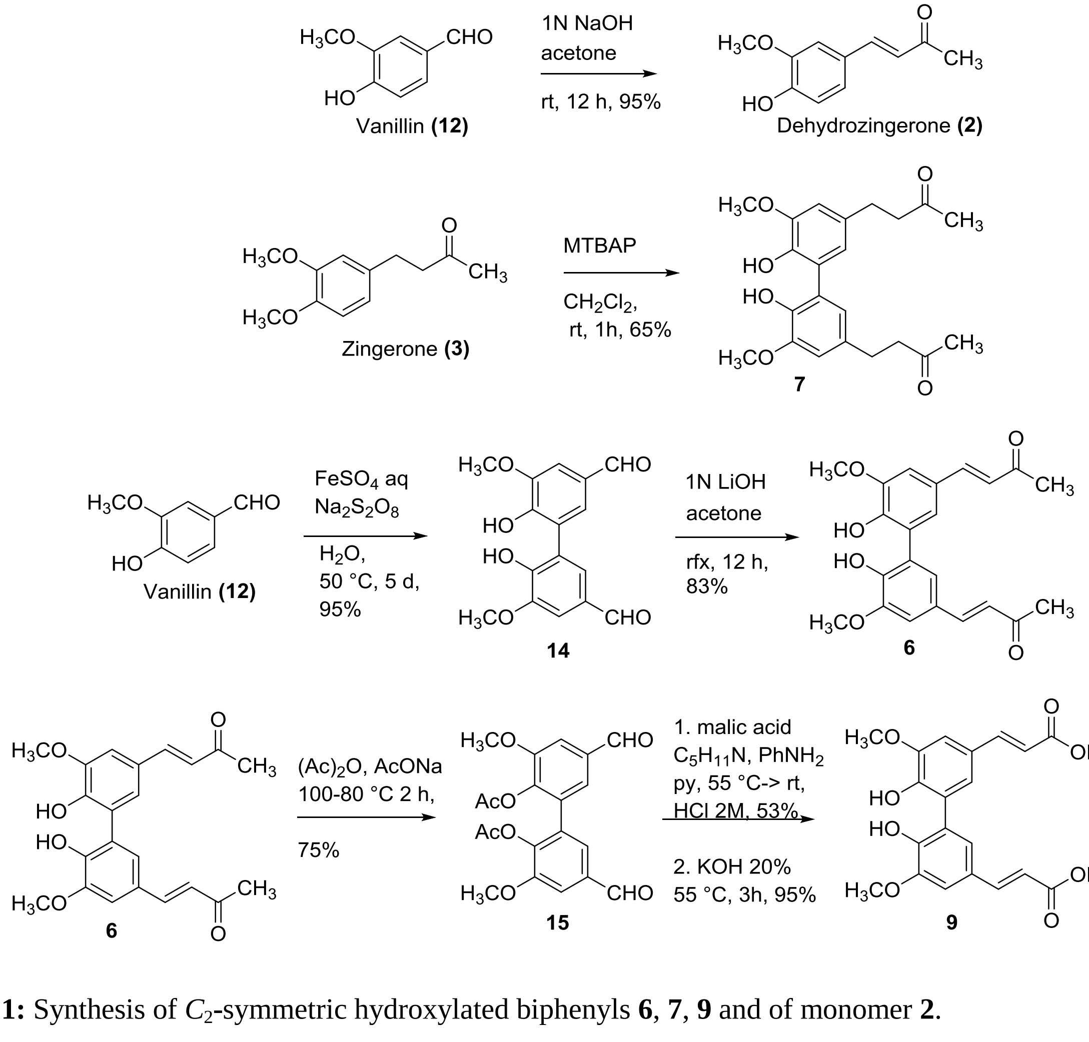

Scheme S1: Synthesis of C_2-symmetric hydroxylated biphenyls **6**, **7**, **9** and of monomer **2**.

1.2. Spectral characteristics of the studied compounds

Dehydrozingerone (**2**). [(*E*)-4-(4-hydroxy-3-methoxyphenyl)but-3-en-2-one]
Mp 126-127°C, ^{1}H NMR δ ppm 2.38 (s, 3H), 3.85 (s, 3H), 5.96 (bs, 1H), 6.53 (d, J=16.0 Hz, 1H), 6.92 (d, J=8.0 Hz, Ar, 1H), 7.04 (d, J=1.6 Hz, Ar, 1H), 7.01 (dd, J=1.6, 8.0 Hz, Ar, 1H), 7.42 (d, J=16.0 Hz, 1H); ^{13}C NMR δ ppm 27.29, 56.93, 109,28, 114.81, 123.95, 124.98, 126.90, 143.77, 146.88, 148.26, 198.46; Anal. Calcd for $C_{11}H_{12}O_3$: C, 68.74; H, 6.29; Found: C, 68.90; H, 6.40.

Vanillin dimer **14**. 6,6'-dihydroxy-5,5'-dimethoxy-[1,1'-biphenyl]-3,3'-dicarbaldehyde
Mp >270°C, ^{1}H NMR (DMSO-*d*$_6$) δ ppm: 3.94 (s, 6H); 7.42 (s, 4H); 9.80 (s, 2H); ^{13}C NMR (DMSO-*d*$_6$) δ ppm: 56.50, 109.70, 125.18, 128.23, 128.62, 148.60, 150.90, 191.60; Anal. Calcd for $C_{16}H_{14}O_6$: C, 63.57; H, 4.67; Found: C, 63.60; H, 4.49.

Dehydrozingerone dimer **6**. [(3*E*,3'*E*)-4,4'-(6,6'-dihydroxy-5,5'-dimethoxy-[1,1'-biphenyl]-3,3'-diyl)bis(but-3-en-2-one)]
Mp 242-243°C, ^{1}H NMR δ ppm 2.36 (s, 6H), 3.98 (s, 6H), 5.30 (bs, 2H), 6.60 (d, J=16.0 Hz, 2H), 7.1 (d, J=2.0 Hz, Ar, 2H), 7.14 (d, J=2.0 Hz, Ar, 2H), 7.47 (d, J=16.0 Hz, Ar, 2H); ^{13}C NMR δ ppm 27.32, 56.22, 108.77, 123.57, 125.27, 125.44, 126.60, 143.51, 145.45, 147.36, 198.30; Anal. Calcd for $C_{22}H_{22}O_6$: C, 69.10; H, 5.80; Found: C, 69.49; H, 5.74.

Zingerone dimer **7**. 4,4'-(6,6'-dihydroxy-5,5'-dimethox-[1,1'-biphenyl]-3,3'-diyl) bis (butan–2-one)]
Mp 85-86°C, ^{1}H NMR δ ppm 2.38 (s, 6H), 2.74-2.88 (series of m, 8h), 3.90 (s, 6H), 6.01 (bs, 2H), 6.71 (d, J=2.0 Hz, 2H), 6.73 (d, J=2.0 Hz, Ar, 2H); ^{13}C NMR δ ppm 29.50, 30.13, 45.46, 56.09, 110.64, 122.68, 124.38, 132.88, 140.90, 147.18, 208.11; Anal. Calcd for $C_{22}H_{26}O_6$: C, 68.38; H, 6.78; Found: C, 68.49; H, 6.74.

Ferulic acid dimer **9**. (2*E*,2'*E*)-3,3'-(6,6'-dihydroxy-5,5'-dimethoxy-[1,1'-biphenyl]-3,3'-diyl)diacrilyc acid
Mp 231°C, ^{1}H NMR (DMSO-*d*$_6$) δ ppm: 3.86 (s, 6H); 6.37 (d, J=16.0 Hz, 2H), 7.01 (d, J=2.0 Hz, 2H), 7.30 (d, J=2.0 Hz, 2H), 7.47 (d, J=16.0 Hz, 2H), 9.03 (bs, 2H); ^{13}C NMR (DMSO-*d*$_6$) δ ppm: 56.48, 109.25, 116.25, 125.25,125.54, 125.64,145.04, 146.89, 148.33, 168.44; Anal. Calcd for $C_{20}H_{18}O_8$: C, 62.17; H, 4.71; Found: C, 62.60; H, 4.49.

2. Studies of antioxidant potential by four models

2.1. Model 1. Estimation of rate constant of antioxidant reaction with peroxyl radicals (k_A) using kinetic chemiluminescence (CL) method

Similarly as described in [s3] the chemiluminescence measurements were performed with the Hamamatsu photosensor module H7467 supplied with the RS-232C interface. The probe chemiluminescent hydrocarbon 'cocktails' for antioxidant assay consisted of chlorobenzene solutions of ethylbenzene (RH) being oxidized by molecular oxygen in the presence of 2,2'-azobisisobutyronitrile (AIBN) as free radical source used to initiate the oxidation process. All the chemicals used in this study were obtained from standard suppliers and purified by known procedures [s4].

Analogous to the description in [s3] the strategy behind the chemiluminescence methodology used in this work is based on the peroxy radical-mediated excited-state generation in a probe solution of a model hydrocarbon substrate (RH, in the present case, ethylbenzene)

upon oxidation of the latter. The advantage of such a kind of hydrocarbon as chemiluminescent substrate resides in a well-understood and straightforward mechanism of its oxidation at moderate temperatures (20–80 °C). Besides, under these conditions, its oxidation rate is very easy to control, simply by choosing an appropriate concentration of the reaction initiator (Y, see Scheme S2), a peroxide or azo compound (in the present case, AIBN), which works as thermal source of initiating free radicals ($Y^{\bullet}$). A relatively low initiation rate of the free radical oxidation process enables the consumption of both the hydrocarbon substrate and the initiator to be neglected during the course of reaction. As a matter of fact, the quasi-stationary reaction conditions allow the chemiluminescence intensity to be kept constant over the time of the experiment in the absence of antioxidative reactants.

The chemiluminescent assay for antioxidant monitoring is based on the competition between the self-reaction of peroxyl radicals (reaction step "k_t" in Scheme S2), giving rise to light emission, and scavenging the peroxyl radicals by antioxidants (monomers [1]and dimers[2]), thus inhibiting the oxidation process and thereby quenching the light emission. The kinetic analysis of the reaction sequence affords suitable expressions which relate to the time profile of the chemiluminescence signal with the pertinent characteristics of an antioxidant being studied. The *strength* of an antioxidant (its most important characteristics in the context of the present work) is quantified by the rate constant k_A, whose value may be acquired from the slope of the chemiluminescence time profile at the inflection point according to Equation 1 [s5,s6] in which

$$(\mathrm{d}i_{rel}/\mathrm{d}t)_{max} = 0.237(k_A/(2k_t)^{0.5})R_{IN} \quad (1)$$

i_{rel} is a dimensionless light intensity given by the ratio of the intensities in the presence (I) and in the absence (I_0) of an antioxidant, $i_{rel} = I/I_0$ (it is noteworthy that consumption of an antioxidant in the probe chemiluminescence solution after the induction period results in recovery of the light-emission intensity, I_∞, i.e., $I_\infty = I_0$ and $i_{rel} = I/I_0 = I/I_\infty$ [s5,s6] and R_{IN} stays for the reaction initiation rate defined by Equation 2. In the latter expression, k_{dec} is the rate constant of the initiator (Y)

$$R_{IN} = 2\gamma_c k_{dec}[Y] \quad (2)$$

decomposition to generate initiating radicals ($Y^{\bullet}$ in Scheme S2) and γ_c is their probability to escape the solvent cage. The $2\gamma_c k_{dec}$ data are available for most of "standard" initiators (such as AIBN, for instance) in a number of organic solvents and in a temperature range of 20 to 80 °C. Calculation of k_{Am} and k_{Ad} from Equation 1 was made taking into account that the value of $2k_t$ obtained experimentally in the same system (chemiluminescence of ethylbenzene) and temperature of 50 °C by Belyakov et al. [s6] is equal to $2k_t = (1.90\pm0.05)\ 10^7\ M^{-1}s^{-1}$.

$Y \rightarrow 2Y^{\bullet}$ (R_{IN})

$Y^{\bullet} + O_2 \rightarrow YO_2^{\bullet}$

$YO_2^{\bullet} + RH \rightarrow YOOH + R^{\bullet}$

$R^{\bullet} + O_2 \rightarrow RO_2^{\bullet}$

$RO_2^{\bullet} + RH \rightarrow ROOH + R^{\bullet}$ (k_p)

---

[1] M_iOH in the kinetic schemes and in the DFT calculation details.

[2] $D_i(OH)_2$ in the kinetic schemes and in the DFT calculation details.

$2RO_2^{\bullet} \rightarrow R{=}O^* + ROH + O_2$ $(2k_t)$

$RO_2^{\bullet} + M_iOH \rightarrow ROOH + M_iO^{\bullet}$ (k_{Am}) in case of monomers

$RO_2^{\bullet} + M_iO^{\bullet} \rightarrow$ *inactive products* (k'_{Am})

$RO_2^{\bullet} + D_i(OH)_2 \rightarrow ROOH + D_i(O^{\bullet})OH$ (k_{Ad}) in case of dimers

$RO_2^{\bullet} + D_i(O^{\bullet})OH \rightarrow ROOH + D_i(O^{\bullet})_2$ (k'_{Ad})

$2RO_2^{\bullet} + D_i(O^{\bullet})_2 \rightarrow$ *inactive products* (k''_{Ad})

Scheme S2: Basic kinetic scheme of initiated oxidation of ethylbenzene (RH) in CL, in absence and in presence of studied compounds.

2.2. Model 2. Estimation of the chain-breaking antioxidant activity of the studied compounds during lipid autoxidation

Lipid sample – similarly as described in [s7] triacylglycerols of commercially available sunflower oil (TGSO) were cleaned from pro- and antioxidants by adsorption chromatography [s8] and stored under nitrogen atmosphere at −20 °C. Fatty acid composition of the lipid substrate was determined according to Christy [s9] by GC analysis of the methyl esters of the total fatty acids obtained with a GC-FID Hewlett-Packard 5890 equipment (Hewlett-Packard GmbH, Austria) and a capillary column HP INNOWAX (polyethylene glycol mobile phase, Agilent Technologies, USA) 30 m × 0.25 mm × 0.25 mm. The temperature gradient started from 165 °C increased to 230 °C with 4 °C/min and held at this temperature for 15 min; injection volume was 1 µL. Injector and detector temperatures were 260 and 280 °C, respectively. Nitrogen was the carrier gas at a flow rate of 0.8 mL/min. The analyses were performed in triplicate. Six different fatty acids were present in TGSO: 16:0 - 6.7%; 18:0 - 3.6%; 18:1 - 25.1%; 18:2 - 63.7%; 20:0 - 0.2%; 22:0 - 0.7%. Lipid samples containing various inhibitors were prepared directly before use. Aliquots of the antioxidant solutions in purified acetone were added to the lipid sample. Solvents were removed under a nitrogen flow.

Lipid autoxidation – analogous to the description in [s7] the process was carried out in a thermostatic bath at 80 °C (± 0.2 °C) by blowing air through the samples in special vessels and was monitored by withdrawing samples at measured time intervals and subjecting them to iodometric determination of the primary products (lipid hydroxyperoxides, LOOH) concentration, i.e., the peroxide value (PV) [s10,s11]. All kinetic data are expressed as the average of two independent measurements.

Triacylglycerols of sunflower oil (TGSO) are a mixture of different types of fatty acids glycerol esters, but only those fatty acids with pentadiene structures are vulnerable to oxidation with atmospheric oxygen. Phenolic antioxidants inhibit or retard lipid oxidation by interfering with either chain propagation or initiation by readily donating hydrogen atoms to lipid peroxyl radicals [s12-s14]. Recently, Kancheva et al. [s15] have published the detailed mechanism of lipid autoxidation in homogeneous solutions under sufficient oxygen pressure in the presence of monomer M_iOH and dimer $D_i(OH)_2$ (Scheme S3).

Basic scheme of lipid (LH) autoxidation

Non-inhibited lipid (LH) autoxidation (in absence of an antioxidant)

Chain generation $2LH + O_2 \rightarrow \delta\, LO_2^{\bullet}$ (R_{IN})

Chain propagation $LO_2^{\bullet} + LH\ (+O_2) \rightarrow LOOH + LO_2^{\bullet}$ (k_p)

Chain termination $LO_2^{\bullet} + LO_2^{\bullet} \rightarrow P$ ($2k_t$)

Chain branching 1 $LOOH\ (+O_2) \rightarrow \delta_1\, LO_2^{\bullet} + P1$ (k_{b1})

Chain branching 2 $LOOH + LH\ (+O_2) \rightarrow \delta_2\, LO_2^{\bullet} + P2$ (k_{b2})

Chain branching 3 $LOOH + LOOH\ (+O_2) \rightarrow \delta_3\, LO_2^{\bullet} + P3$ (k_{b3})

Inhibiting reactions, responsible to the antioxidant activity of monomers M_iOH

Chain termination with H atom transfer reaction $LO_2^{\bullet} + M_iOH \rightarrow LOOH + M_iO^{\bullet}$ (k_{Am})

Chain termination with cross-recombination $LO_2^{\bullet} + M_iO^{\bullet} \rightarrow$ inactive products (k'_{Am})

Chain termination with homo-recombination $2M_iO^{\bullet} \rightarrow$ inactive products (k_{tMO})

Side reactions of $M_iO^{\bullet}$, decreasing the antioxidant activity of M_iOH

Reverse inhibition reaction $M_iO^{\bullet} + LOOH \rightarrow LO_2^{\bullet} + M_iOH$ (k_{-Am})

Additional propagation reaction $M_iO^{\bullet} + LH\ (+O_2) \rightarrow \delta_3\, LO_2^{\bullet} + M_iOH$ (k_{pMO})

Quinoidal peroxides (QP) formation $2M_iO^{\bullet} + O_2 \rightarrow QP$ (k_{QP})

Quinoidal peroxides (QP) decomposition $QP\ (+ LH, + O_2) \rightarrow \delta_4\, LO_2^{\bullet} + P_4$ (k'_{QP})

In this mechanism: LH is linoleic acid with its allylic hydrogen; M_iOH - the studied monomeric antioxidant; $LO_2^{\bullet}$ - lipid peroxide radicals; $M_iO^{\bullet}$- phenoxyl radicals; P , P_A, P_1—P_4 – corresponding products formed; QP- quinoidal peroxides; R_{IN} - the rate of initiation; k_p, k_t, k_b, k_{Am}, k'_{Am} , k_{tMO}, k_{pMO}, k_{QP} – corresponding rate constants of different reactions.

In the presence of an antioxidant, the rate of hydroperoxides formation is related to the ratio of the lipid [LH] concentration to that of the antioxidant [M_iOH] concentration.

The classical rate laws for both uninhibited (R_c) and inhibited (R_A) lipid autoxidation, assuming the long chain approximation, are given by Eqs 3 and 4:

Rate of non-inhibited oxidation (R_c) $R_c = k_p\,[LH](R_{IN}/k_t)^{0.5}$ (3)

Rate of inhibited oxidation (R_{Am}) $R_{Am} = k_p[LH]R_{IN}/nk_{Am}[M_iOH]_0$ (4)

Inhibiting reactions, responsible to the antioxidant activity of the dimer $D_i(OH)_2$

Chain termination with H-atom transfer reaction:

$D_i(OH)_2 + LO_2^{\bullet} \rightarrow D_i(OH)O^{\bullet} + LOOH$ (k_{Ad}) – the key reaction of inhibited lipid autoxidation

$D_i(OH)O^{\bullet} + LO_2^{\bullet} \rightarrow D_i(O^{\bullet})_2 + LOOH$ (k'_{Ad}) [$D_i(O^{\bullet})_2$ –biradical of $D_i(OH)_2$; active intermediate]

Chain termination with cross recombination reaction:

$D_i(OH)O^{\bullet} + LO_2^{\bullet} \rightarrow D_i(OH)OL + O_2$ (active intermediate with one free OH group)

$D_i(O^{\bullet})_2 + 2LO_2^{\bullet} \rightarrow$ inactive products (k''_{Ad})

Chain termination with homo recombination reaction:

$2\,D_i(OH)O^{\bullet} \rightarrow [D_i(OH)O]_2$ - active intermediate with 2 free OH groups

Additional chain termination with H atom transfer reaction:

$D_i(OH)OL + LO_2^{\bullet} \rightarrow LOOH + D_i(O^{\bullet})OL$ - active intermediate

$[D_i(OH)O]_2 + 2LO_2^{\bullet} \rightarrow 2LOOH +$ inactive products

Additional cross-recombination reaction:

$D_i(O^{\bullet})OL + LO_2^{\bullet} \rightarrow$ inactive product

Additional homo-recombination reaction:

$2\,D_i(O^{\bullet})OL \rightarrow$ inactive product

Scheme S3: Basic kinetic scheme of uninhibited and inhibited (in presence of monomers and dimers) lipid autoxidation.

Rate of inhibited oxidation (R_{Ad}) $R_{Ad} = k_p[LH]R_{IN}/nk_{Ad}[D_i(OH)_2]_0$ (5)
In Equation 3-5: $k_p/(2k_t)^{0.5}$ is the oxidizability of the lipid substrate (linoleic acid glycerol ester); n- is the number of radicals trapped per inhibitor for molecule (the stoichiometric factor).

Determination of the main kinetic parameters of the studied compounds [s12-s15]

Antioxidant efficiency means the potency of an antioxidant to increase the persistence towards oxidation of a lipid substrate by blocking the radical-chain process. It was presented with a protection factor (PF) meaning how many times the antioxidant increases the persistence against oxidation of the lipid sample, determined as a ratio between the induction periods in presence (IP_A) and in absence (IP_C) of an antioxidant, i.e., PF = IP_{Am}/IP_C for the monomers and PF = IP_{Ad}/IP_C for the dimers.

Inhibition degree (ID) is a measure of the antioxidant reactivity, which manifests how many times the antioxidant shortens the oxidation chain length, i.e. ID = R_C/R_{Am} in the case of monomers and ID = R_C/R_{Ad} in the case of dimers. For that reason, it is one of the most important kinetic parameters. Initial rates of lipid autoxidation in the absence (R_C) and in the presence of the antioxidant (R_{Am} or R_{Ad}) were found from the tangent at the initial phase of the kinetic curves of hydroperoxides accumulation.

Chain length of non-inhibited oxidation (ν_0)	$\nu_c = R_c/R_{IN}$
Chain length of inhibited oxidation (ν_{Am} or ν_{Ad})	$\nu_{Am} = R_{Am}/R_{IN}$ and $\nu_{Ad} = R_{Ad}/R_{IN}$
Inhibition degree (ID)	
In case of monomers	ID = $R_c/R_{Am} = \nu_c/\nu_{Am}$
In case of dimers	ID = $R_c/R_{Ad} = \nu_c/\nu_{Ad}$

Statistical analysis of the induction period (IP) determination. Ten independent experiments were carried out in association with the previous results on inhibited oxidation. The standard deviation (SD) for different mean values of IP was (in h): IP = 2.0, SD = 0.2; IP = 5.0, SD = 0.3; IP = 15.0, SD = 1.0; IP = 25, SD = 1.5; IP = 50.0, SD = 3.0. The SD of PV determination (in meq/kg), according to the modified iodometric method for different mean values of PV, was: PV = 12.0, SD = 1.0; PV = 30.0, SD = 2.0; PV = 70.0, SD = 5.0; PV = 150.0, SD = 10; PV = 250.0, SD = 20. The R_A and R_C were nearly constant varying by less than 2 %.

2.3.Model 3. Estimation of oxygen radical absorbance capacity (ORAC)

ORAC was measured according to the method of Ou et al. [s16] with some modifications, analogous to the description in [s17]. The method affords the antioxidant scavenging activity against peroxyl radical generated by *2,2'*-azobis (*2*-amidinopropane) dihydrochloride (AAPH, Y_A) at 37 °C. Fluorescein[3] was used as the fluorescent probe. AAPH and fluorescein were obtained from Sigma-Aldrich Chemie (Steinheim, Germany). The experiments were performed on FLUOstar OPTIMA fluorimeter (BMG LABTECH, Offenburg, Germany). The loss of fluorescence of fluorescein was an indication of the extent of fluorescein damage in its reaction with the peroxyl radical. The protective effect of an antioxidant was measured by assessing the area under the fluorescence decay curve (AUC) as compared to that of blank in which no

[3] FLOH in the kinetic scheme S4.

antioxidant is present. Solutions of AAPH, fluorescein and Trolox were prepared in a phosphate buffer (75 mM, pH 7.4). Samples were diluted in phosphate buffer as well. Reaction mixture (total volume 200 μL) contained fluorescein (170 μL, final concentration 5.36×10^{-8} M), AAPH – (20 μL, final concentration 51.51 mM), and sample (10 μL). Fluorescein solution and sample were incubated at 37 °C for 20 min, and AAPH (dissolved in 37 °C buffer) was added. The mixture was incubated for 30 s before the initial fluorescence was measured. After that, the fluorescence readings were taken at the end of every cycle after shaking. For the blank, 10 μL of phosphate buffer was used instead of the sample. Antioxidant activity was expressed in Trolox equivalents. For defining the standard curve 10 μL of 3.13, 6.25, 12.50, 25 and 50 μM Trolox solutions (final concentrations 0.16, 0.31, 0.63, 1.25 and 2.50 μM, respectively) were used instead of the sample. One ORAC unit is assigned to the net protection area, provided by 10 μL, 1 μM Trolox solution (final concentration – 0.05 μM). Results were expressed as Relative Trolox Equivalents (RTE).

According to the basic kinetic consideration of Huang et al. [s18] the new reaction sequence (Scheme 4) is proposed:

$Y_A \rightarrow 2\,Y_A^{\bullet}$	(R_{IN})	
$Y_A^{\bullet} + O_2 \rightarrow Y_AO_2^{\bullet}$		
$Y_AO_2^{\bullet} + FLOH \rightarrow Y_AOOH + FLO^{\bullet}$	(k_{FL})	
$Y_AO_2^{\bullet} + FLO^{\bullet} \rightarrow$ *inactive products*	(k'_{FL})	
$Y_AO_2^{\bullet} + M_iOH \rightarrow Y_AOOH + M_iO^{\bullet}$	(k_{Am})	in case of monomers
$Y_AO_2^{\bullet} + M_iO^{\bullet} \rightarrow$ *inactive products*	(k'_{Am})	
$Y_AO_2^{\bullet} + D_i(OH)_2 \rightarrow Y_AOOH + D_i(O^{\bullet})OH$	(k_{Ad})	in case of dimers
$Y_AO_2^{\bullet} + D_i(O^{\bullet})OH \rightarrow Y_AOOH + D_i(O^{\bullet})_2$	(k'_{Ad})	
$2\,Y_AO_2^{\bullet} + D_i(O^{\bullet})_2 \rightarrow$ *inactive products*	(k''_{Ad})	

Scheme S4: Basic kinetic scheme of initiated oxidation in ORAC.

The net AUC was obtained by subtracting the AUC of the blank from that of the sample. The relative ORAC value (Relative Trolox Equivalents) was calculated as [s16]:

$$[(AUC_{Sample} - AUC_{Blank})/(AUC_{Trolox} - AUC_{Blank})] \times (\text{molarity of Trolox/molarity of sample}) \quad (6).$$

2.4. Model 4. DFT calculations

In this work, DFT calculations have been performed using GAUSSIAN 09 program package [s19]. The geometries of curcumin, hydroxylated biphenyls **6**–**9**, corresponding monomers **2**–**5**, fluorescein, DL-α-tocopherol, Trolox and their radicals were optimized using unrestricted open-shell approach (UB3LYP) and 6-31+G(d,p) basis sets [s20-s22] without symmetry constraints (C_1 symmetry was assumed) with the default convergence criteria. The reliability of the used combination of the above-mentioned hybrid functional and double-zeta basis set with polarization and diffusion functions in the description of the phenoxyl radicals was proved by Anouar et al. for ferulic acid [s23]. No spin contamination is found for radicals. Local minima were verified by establishing that the Hessians have zero negative eigenvalues. Unscaled thermal corrections to enthalpy were added to the total energy values. The homolytic bond dissociation enthalpy (BDE) was used like descriptor of the free-radical scavenging activity. The BDEs for the generation of the respective radicals/biradicals from the parent compounds are

calculated by the following formula: in case of monomers (M_iOH) BDE(r) = $H_{298}(M_iO^\bullet) + E_T(H^\bullet) - H_{298}(M_iOH)$, in case of dimers $[D_i(OH)_2]$ BDE(r) = $H_{298}[D_i(O^\bullet)OH] + E_T(H^\bullet) - H_{298}[D_i(OH)_2]$, and BDE(br) = $H_{298}[Di(O^\bullet)_2] + E_T(H^\bullet) - H_{298}[D_i(O^\bullet)OH]$, where $H_{298}(M_iO^\bullet)$, $H_{298}[D_i(O^\bullet)OH]$, $H_{298}[D_i(O^\bullet)_2]$, $H_{298}(M_iOH)$ and $H_{298}[D_i(OH)_2]$ are the B3LYP calculated enthalpies at 298 K for radical species $M_iO^\bullet$ and $D_i(O^\bullet)OH$, biradical species $D_i(O^\bullet)_2$ and neutral molecules M_iOH and $D_i(OH)_2$, and $E_T(H^\bullet)$ (calculated total energy of $H^\bullet$) is −313.93 kcal·mol^{-1} (−0.5002728 a.u.). The integral equation formalism (IEF) of the polarizable continuum model (PCM) [s24,s25] was applied in order to take into account the solvent effect (water).

References

s1. Marchiani, A.; Mammi, S.; Siligardi, G.; Hussain, R.; Tessari, I.; Bubacco, L.; Delogu, G.;. Fabbri, D.; Dettori, M.A.; Sanna, D.; Dedola, S.; Serra, P.A.; Ruzza, P. *Amino Acids*, **2013,** *45,* 327-338.

s2. Russel, W. R.; Scobbie, L.; Chesson, A. *Bioorg.& Med. Chem.* **2005,** *13,* 2537-2546.

s3. Fedorova, G. F.; Menshov, V. A.; Trofimov, A. V.; Vasil'ev, R. F., *Analyst* **2009,** *134,* 2128–2134.

s4. Belyakov, V. A.; Vasilev, R. F.; Fedorova, G. F.; *Kinet. -Catal.* **1996,** *37,* 542-552.

s5. Fedorova, G. F.; Trofimov, A. V.; Vasil'ev, R. F.; Veprintsev, T. L. *ARKIVOC* **2007,** *01,* 163-215.

s6. Belyakov, V. A.; Vasilev, R. F.; Fedorova, G. F. *Kinet. -Catal.* **2004,** *45,* 329-336.

s7. Kancheva, V. D., Saso, L., Angelova, S., Foti, M.C., Slavova-Kasakova, A., Daquino, C., Enchev, V., Firuzi,O., Nechev, J. *Biochimie*, **2012**, *94*, 403-415.

s8. Popov, A.; Yanishlieva, N.; Slavcheva, J. *Compt. Rend. Acad. Bulg. Sci.* **1968,** *21,* 443-446.

s9. Christie, W. W. *Lipid Analysis*, 3rd ed.; The Oily Press, Barnes & Associates, Bridgwater: England, 2003.

s10. Yanishlieva, N. V.; Popov, A. D.; Marinova, E. M. *Compt. Rend Acad. Bulg. Sci.* **1978,** *31,* 869-871.

s11. Kancheva, V. D. *Eur. J. Lipid Sci.Technol.* **2009,** *111,* 1072-1089.

s12. E.N. Frankel, *Lipid oxidation*. Dundee, Scotland: The Oily Press, 1998.

s13. Denisov, E. T.; Denisova, T. G. *Chem. Rev*. **2009,** *78,* 1129-1143.

s14. Yanishlieva-Maslarova, N.; Inhibiting oxidation. In: *Antioxidants in Food. Practical Applications*; J. Pokorny, Yanishlieva, N.; Gordon M., Eds; Cambridge, UK: CRC Press, Boca Ration, Woodhead Publishing Ltd. 2001, pp. 22-70.

s15. Kancheva, V. D.; Slavova-Kazakova, A.; Fabbri, D.; Dettori, M. A.; Delogu, G.; Janiak, M.; Amarowicz, R. *Food Chem* **2014,** *157,* 263-274.

s16. Ou, B.; Hampsch-Woodill, M.; Prior R. L., *J. Agric. Food Chem.* **2001,** *49,* 4619-4626.

s17. Soare, L.C.; Ferdes, M.; Stefanov, S.; Denkova, Z.; Nicolova, R., Denev, P.; Ungureanu,C. A. Rev.Chim.(Bucharest), **2012**, *63*, 432-434.

s18. Huang, D.; Ou, B.; Prior, R. L. *J. Agric. Food Chem.* **2005,** *53,* 1841-1856.

s19. Gaussian 09, Revision C.01, Gaussian, Inc., Wallingford CT, 2010.

s20. Hehre, W. J.; Ditchfield, R.; Pople, J. A. *J. Chem. Phys.*, **1972,** *56,* 2257-2261.

s21. Clark, T.; Chandrasekhar, J.; Spitznagel, G. W.; Schleyer, P. v.R. Li-F. *J. Comp. Chem.* **1983,** *4,* 294-301.

s22. Frisch, M. J.; Pople, J. A.; Binkley, J. S. *J. Chem. Phys.* **1984,** *80,* 3265-3269.

s23. Anouar, E.; Kosinová, P.; Kozlowski, D.; Mokrini, R.; Duroux, J. L.; Trouillas, P. *Phys. Chem. Chem. Phys.* **2009,** *11,* 7659-7668.

s24.Cammi, R.; Cossi, M.; Tomasi, J. *J. Chem. Phys.* **1996,** *104,* 4611-4620.

s25.Tomasi, J.; Cammi, R.; Mennucci, B.; Cappelli, C.; Corni, S. *Phys. Chem. Chem. Phys.* **2002,** *4,* 5697-5712.
